# Potential regenerative treatment strategies for intervertebral disc degeneration in dogs

**DOI:** 10.1186/1746-6148-10-3

**Published:** 2014-01-04

**Authors:** Frances C Bach, Nicole Willems, Louis C Penning, Keita Ito, Björn P Meij, Marianna A Tryfonidou

**Affiliations:** 1 Department of Clinical Sciences of Companion Animals, Faculty of Veterinary Medicine, Utrecht University, Utrecht, Netherlands; 2 Department of Biomedical Engineering, Eindhoven University of Technology, Eindhoven, Netherlands; 3 Department of Orthopaedics, University Medical Center Utrecht, Utrecht, Netherlands

**Keywords:** Intervertebral disc, Degeneration, Spine, Dog, Regenerative medicine, Mesenchymal stem cell, Notochordal cell

## Abstract

Pain due to spontaneous intervertebral disc (IVD) disease is common in dogs. In chondrodystrophic (CD) dogs, IVD disease typically develops in the cervical or thoracolumbar spine at about 3–7 years of age, whereas in non-chondrodystrophic (NCD) dogs, it usually develops in the caudal cervical or lumbosacral spine at about 6–8 years of age. IVD degeneration is characterized by changes in the biochemical composition and mechanical integrity of the IVD. In the degenerated IVD, the content of glycosaminoglycan (GAG, a proteoglycan side chain) decreases and that of denatured collagen increases. Dehydration leads to tearing of the annulus fibrosus (AF) and/or disc herniation, which is clinically characterized by pain and/or neurological signs. Current treatments (physiotherapy, anti-inflammatory/analgesic medication, surgery) for IVD disease may resolve neurological deficits and reduce pain (although in many cases insufficient), but do not lead to repair of the degenerated disc. For this reason, there is interest in new regenerative therapies that can repair the degenerated disc matrix, resulting in restoration of the biomechanical function of the IVD. CD dogs are considered a suitable animal model for human IVD degeneration because of their spontaneous IVD degeneration, and therefore studies investigating cell-, growth factor-, and/or gene therapy-based regenerative therapies with this model provide information relevant to both human and canine patients. The aim of this article is to review potential regenerative treatment strategies for canine IVD degeneration, with specific emphasis on cell-based strategies.

## Introduction

**Figure 1:**
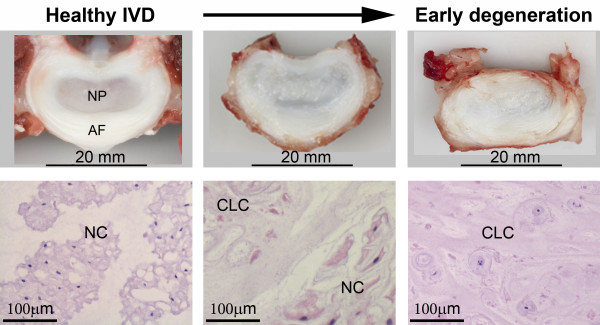
**The transition from a healthy to an early degenerated intervertebral disc.** Macroscopic and microscopic images showing a healthy intervertebral disc (IVD) (left), and the transition (middle) into an early degenerated IVD (right). The healthy IVD (upper left) consists of a well-distinguished lamellar annulus fibrosus (AF) and a bean-shaped nucleus pulposus (NP). During disc degeneration, the distinction between AF and NP becomes less clear (upper middle and right). In the healthy NP, notochordal cells (NCs) are the main cell type present (lower left), whereas in the transitional NP, a mixture of cartilage-like cells (CLCs) and NCs is present (lower middle). In early IVD degeneration, CLCs are the main cell type present in the NP (lower right).

The intervertebral disc (IVD) arises from the embryonic notochord and mesenchyme and develops into a complex tissue that permits movement between vertebrae and provides flexibility and integrity to the spine [1]. The IVD consists of the vertebral endplates (EPs), the annulus fibrosus (AF), and the nucleus pulposus (NP) (Figure 1). The AF contains concentric lamellae mainly composed of collagen type I, elastin fibres, and fibroblast-like cells. Together with the hyaline cartilage EPs, it encloses the bean-shaped NP [2,3]. By enabling diffusion and permeability, the EPs play an essential role in NP nutrient delivery, since the latter has no direct blood supply [2]. The NP contains a highly hydrated gelatinous matrix, mainly composed of proteoglycan and collagen type II [2]. Cations, which are attracted to the negatively charged proteoglycans, create a strong osmotic gradient that draws water molecules into the NP [2]. During early development, the NP contains a relatively large amount of cells and a small amount of extracellular matrix (ECM), whereas the ECM/cell ratio is high in the mature NP [3,4].

IVD degeneration is characterized by changes in the biochemical composition and mechanical integrity [2,4]. The glycosaminoglycan (GAG, a proteoglycan side chain) content decreases and catabolic matrix metalloproteinase (MMP) activity and denatured collagen content increases - the latter creating a more rigid IVD matrix reviewed in [2,5,6]. Matrix repair is impaired in the avascular IVD, resulting in weakening and increased vulnerability to damage by physiologic loading. A vicious circle of continuous damage and inadequate repair develops, leading to disc degeneration rather than healing. Once the degenerative process starts, it is progressive [7]. The decrease in GAG content, and subsequent dehydration, causes a decrease in disc height [8]. Compressive forces cause increased loading on the AF, generating a concave bending load on the EPs. This change in the direction of loading forces is considered to be responsible for AF tearing, EP fracturing, and/or disc herniation. Herniation usually occurs on the dorsal side, because the ventral part of the AF is two to three times thicker than the dorsal part and because the vertebral column is normally more flexed than extended, resulting in higher tension in the dorsal part than in the ventral part [2].

Many dogs suffer from the clinical consequences of IVD degeneration, and IVD disease is a relatively common reason for euthanasia [9]. IVD degeneration impairs the function of the spinal unit (IVD, EPs, ligaments, facet joints and vertebral body), causing secondary osteoarthritic changes, bone sclerosis/spondylosis, and neurological signs and deficits due to spinal cord or nerve root compression [10]. Owners of dogs may report unilateral or bilateral lameness or paresis/paralysis, toe dragging, low tail or neck carriage, difficulties with rising, sitting or lying down, reluctance to jump or climb, urinary or faecal incontinence, and hyperesthesia or self-mutilation [11]. Furthermore, pain is evoked by pressure applied to the affected spinal region during the clinical examination by veterinary specialists [11]. However, IVD degeneration can also be asymptomatic.

Dog breeds can be divided into chondrodystrophic (CD) and non-chondrodystrophic (NCD) breeds based on their physical appearance. CD dog breeds (e.g. Beagles and Dachshunds) have short bowlegs because of disrupted endochondral ossification. This trait has strongly been linked with IVD degeneration, which is considered to be polygenetic [5]. In CD dog breeds, IVD disease (mainly Hansen type I herniation) typically develops in the cervical (C2-C6) or thoracolumbar (T11-L3) spine at about 3–7 years of age [5]. NCD dog breeds, especially large breeds, can also develop IVD disease (mainly Hansen type II herniation), but in the caudal cervical (C5-T1) or lumbosacral (L6-S1) spine at about 6–8 years of age, mostly due to trauma or “wear and tear” [5,9]. The macroscopic, histopathological, and biochemical changes as well as the diagnostics and treatment of IVD disease are rather similar in NCD and CD dogs [5,9].

Current treatments for IVD disease in dogs focus on alleviating pain and include physiotherapy, anti-inflammatory/analgesic medication, and surgery. The aim of surgery for IVD disease is to relieve the compression of neural structures, and procedures include removal of the NP (nucleotomy) through fenestration of the AF alone or combined with partial removal of the vertebral roof (laminectomy) or vertebral body (ventral decompression) [12]. Above mentioned therapies may resolve neurological deficits and reduce pain (although in many cases insufficient), but they do not lead to repair of the degenerated IVD. In fact, long-term medication can cause side-effects, surgery can lead to spinal instability and adjacent segment disease, and recurrence of IVD disease may occur [12]. Therefore, there is increasing interest in regenerative therapies aimed at the biological repair of the degenerated IVD, including cell-based strategies and the use of growth factors or gene therapy. The specific aim of these therapies is to repair the degenerated disc matrix and in this way restore the biomechanical function of the IVD [13,14]. Research in the field of regenerative medicine increases our understanding of disease processes and findings may ultimately be translated into therapeutic interventions for veterinary patients. The focus of this review is to discuss potential regenerative treatment strategies for canine IVD degeneration, with specific emphasis on cell-based strategies.

## Models to develop new regenerative treatment strategies for IVD degeneration

**Figure 2:**
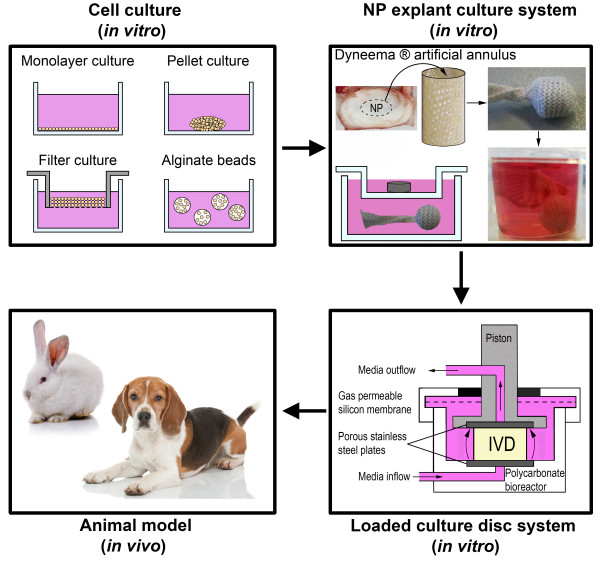
**Regenerative treatment strategies for intervertebral disc (IVD) degeneration.** Upper left: *in vitro* cell cultures: monolayer (2D), pellet culture (3D), filter culture (3D), and cell-containing alginate beads (3D). Upper right: the *in vitro* nucleus pulposus (NP) explant culture system, in which the NP is cultured in an artificial annulus system. As the artificial annulus may be buoyant, a stainless steel cylinder is added to keep the NP submerged. Lower right: the *in vitro* loaded culture disc system, in which intact IVD explants are cultured under loading conditions with preservation of biological and structural integrity. Lower left: *in vivo* animal models, e.g., the rabbit, in which IVD degeneration is often experimentally induced, and the Beagle (chondrodystrophic dog breed), which develops spontaneous IVD degeneration from 1 year of age (right). Pictures of the rabbit and Beagle are obtained from Depositphotos (http://depositphotos.com).

Regenerative treatment options are developed *in vitro* and then extrapolated to *in vivo* animal models before being used in the clinic. Commonly used *in vitro* cell culture models are 2D monolayer and 3D cell pellet, filter, or alginate bead cell culture (Figure 2). A major disadvantage of these *in vitro* models, however, is that the native tissue environment is lost during cell isolation, which might affect cell behaviour [15]. Thus cell culture experiments may not adequately represent the *in vivo* situation, and care should be taken when interpreting the results of such studies. For this reason, tissue explant systems have been developed, in which an environment is created that resembles the *in vivo* situation [16]. However, a drawback of NP explants is that swelling pressure is no longer balanced by the AF and hyperosmolarity within the NP is difficult to maintain, which results in NP tissue swelling. A special fibre jacket (artificial annulus) has been developed to mimic the AF in the so-called NP explant system, creating a physiological model to test the efficacy of regenerative therapies for IVD degeneration [15,17]. To mimic the *in vivo* situation even closer, whole organ culture bioreactor systems, e.g. the loaded culture disc system, have been developed, in which intact IVD explants, including AF, NP, and EPs, are kept alive under loading conditions for as long as 6 weeks with preservation of biological and structural integrity [18,19]. The last step for validating regenerative medicine treatment strategies involves the use of *in vivo* animal models. Models based on mice, rats, rabbits, goats, and dogs with experimentally induced IVD degeneration have often been used [20], but these animals, with the exception of dogs, do not spontaneously develop IVD degeneration and disease. Interestingly, some *in vivo* IVD regenerative studies have been performed with laboratory dogs with experimentally induced IVD degeneration [21-26]. To our knowledge, dogs with naturally occurring IVD degeneration have not been used, even though it is recognized that CD dog breeds are a suitable animal model of spontaneous IVD degeneration [9,13].

## Regenerative treatment options

### Cell-based therapies

**Figure 3:**
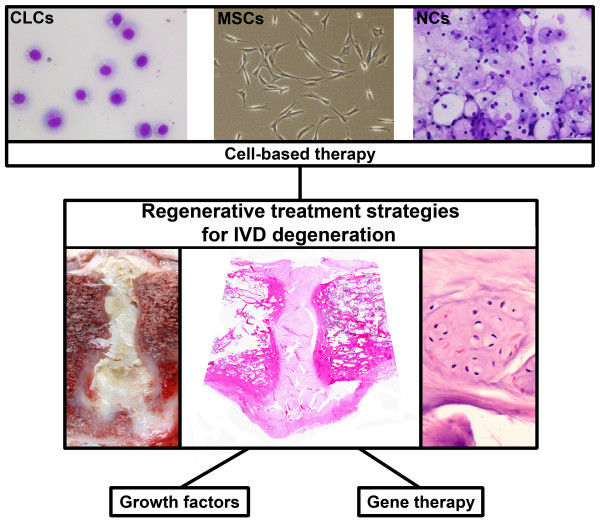
**Regenerative treatment options for intervertebral disc (IVD) degeneration: cell-based therapy, growth factors, and gene therapy. **Cell-based treatment strategies have mainly used chondrocyte-like cells (CLCs), mesenchymal stromal (stem) cells (MSCs), and notochordal cells (NCs). The lower images show a severely degenerated IVD macroscopically (left) and on haematoxylin & eosin staining (middle and right). The lower right image shows a cluster of CLCs, which are frequently observed in (severely) degenerated IVDs.

The synthesis of NP matrix can be stimulated by means of growth factors and/or gene therapy. However, since there are relatively few cells in the degenerated IVD and cell viability is impaired, stimulation of the remaining cells may be insufficient to achieve repair [13]. Cell-based therapies may overcome this problem (Figure 3). Thus far, cell-based treatment strategies have mainly used chondrocyte-like cells (CLCs), mesenchymal stromal (stem) cells (MSCs), and notochordal cells (NCs) [13]. These cell types are discussed below with regard to their characteristics, effectiveness, benefits, and drawbacks.

#### Chondrocyte-like cells (CLCs)

The cells found in the degenerating NP are similar, but not identical, to articular chondrocytes and are therefore termed chondrocyte-like cells (CLCs) [27]. Insertion of autologous and allogeneic CLCs in the NP has been shown to retard IVD degeneration in various species without inducing any appreciable host-versus-graft response [21,28,29]. This is in line with the notion that the IVD is immune privileged due to its avascularity [30] and the expression of Fas ligand (FasL), which induces apoptosis of invading Fas-bearing T-cells [24,31].

Although CLCs have regenerative potential, the severity of IVD degeneration can limit their regenerative capacity. Degenerated CLCs may lose specific characteristics (e.g. their chondrogenic potential), making them less suitable for tissue engineering purposes. CLC transplantation has its limitations because these cells can only be obtained from herniated discs, cell numbers are low, and extensive expansion before transplantation is needed [32]. Preconditioning expanding CLCs with AF cells, however, improved the performance of CLCs in a rabbit IVD degeneration model [33]. Also, co-culture of CLCs with MSCs enhanced the biological activity and viability of CLCs *in vitro*, as evidenced by increased cell proliferation and matrix synthesis [32,34-39]. The latter is consistent with the well-known beneficial effect of MSCs on articular chondrocytes [40].

#### Mesenchymal stromal (stem) cells (MSCs)

NP-derived cells are of limited availability [41] and, therefore, current regenerative strategies focus on stem cells, particularly MSCs. MSCs are emerging as a leading cellular therapy for several diseases, since they can easily be isolated from a variety of tissues, including bone marrow, adipose and synovial tissue, muscle, placenta, and umbilical cord blood. MSCs can differentiate into different cell types, such as osteoblasts, adipocytes, chondrocytes, myocytes, and neural cells [42,43], depending on their environment [7]. In addition, MSCs have immunosuppressive properties and secrete trophic/growth factors (anti-apoptotic, stimulation of proliferation and differentiation) that support regenerative processes [43-45]. The unique combination of these properties makes MSCs highly suitable for tissue replacement therapies. Bi-directional intercellular secretion and transfer of membrane-bound components (e.g. microvesicles) has been proposed as a mechanism of cellular communication between MSCs and CLCs, and not cell fusion or gap-junctional communication. In this way, soluble, cellular or nuclear (e.g. functional gene) components can be transferred between the two cell types, causing phenotypic alterations [46].

*In vivo* injected MSCs can maintain their viability and proliferate within the IVD [47]. Bone marrow-derived stem cell (BMSC) transplantation was found to counteract IVD degeneration and/or increase the proteoglycan content of IVDs in experimental rabbit and mouse models [7,30,48-51]. Adipose-derived stem cell (ASC) and BMSC delivery has also been shown to promote disc regeneration in an experimentally induced IVD degeneration in (Beagle [24]) dogs [25]. In addition, MSCs may be responsible for sustaining the IVD immune privilege by differentiating into cells expressing FasL [24]. The optimal number of BMSCs for intradiscal delivery (4 weeks after NP aspiration) was estimated in Beagles, based on the survival rate and apoptosis of CLCs in BMSC-injected IVDs [26]. In IVDs injected with 10^6^ MSCs, the microenvironment and ECM abundance was maintained, whereas the injection of fewer cells (10^5^ MSCs) resulted in fewer viable cells and the injection of more cells (10^7^ MSCs) resulted in more apoptotic cells [26].

Despite the promising experimental studies and clinical trials, there is uncertainty about the chondrogenic differentiation of MSCs. Can MSCs differentiate into hyaline articular chondrocytes instead of CLCs? MSCs have been reported to undergo chondrocytic differentiation and form repair tissue that resembles hyaline cartilage during *in vitro* studies or after transplantation into the NP [30,52]. Morphologically, articular cartilage and NP tissue consist of chondrocytes surrounded by ECM, but there are considerable differences in composition and biomechanical function [53]. However, properly differentiated MSCs express chondrogenic cell marker genes and have a proteoglycan:collagen ratio closer to that of NP tissue than that of hyaline articular cartilage [53-55].

Recently, healthy and degenerated NPs of various species, including dogs, have been reported to contain multipotent NP progenitor cells (NPPCs) [10,27,56,57]. These NPPCs express genes typical for stem cells, but differ from MSCs in the higher expression of the *Nanog* “stemness” gene [10]. NPPCs are able to differentiate into the chondrogenic lineage *in vitro*, and to survive in the aneural, avascular, hypoxic NP [10,14]. Thus, NPPCs might be appropriate for use in regenerative strategies to treat IVD degeneration.

#### Notochordal cells (NCs)

The notochord is a mesodermal, rod-like structure that defines the primitive axis of the body and serves as the centre of development of the axial skeleton during embryogenesis. Notochordal signals generate a mesenchymal peri-notochordal sheath, which eventually gives rise to the AF, EPs, and vertebral bodies [8]. The notochord condenses within the primitive AF to form the NP [1,4]. The composition of the NP changes as the IVD matures: the number of cells of notochordal origin decreases and the smaller CLCs increase in number [4] (Figure 1). NCs are characterized by their morphology: they are large and have cytoplasmic vesicles, the content and function of which are still debated [8]. NCs are usually found in clusters and secrete matrix rich in proteoglycan and collagen type II [2]. They have considerable regenerative potential and restorative capacity for other cells (CLCs and MSCs), which makes them an interesting focus for regenerative strategies.

Loss of the NC population is associated with the early development of IVD disease [58], and thus restoring the NC population may help to delay or even reverse IVD degeneration [14]. The NC population undergoes species-specific changes [4,8]. Most studies report that in humans, NCs disappear when they are about 7–10 years of age, whereas the age of onset of disc degeneration is 30–50 years [8]. This means that CLCs also reside in healthy human IVDs. However, in dogs, NCs are replaced by CLCs at about 1 year of age in CD breeds, but remain the predominant cell type until middle/old age in NCD breeds [4,5,14]. In general, NCs are not present in degenerated canine IVDs.

Interestingly, intradiscal MSC injection increased the number of NCs and matrix deposition in murine degenerated IVDs [30]. This suggests that MSCs can promote NC proliferation and/or prevent NC apoptosis and that the anabolic NC function can be stimulated by MSCs. Taking into consideration the recently described NPPC population (which shows stem cell characteristics) in healthy and degenerated IVDs, it is tempting to hypothesize that NPPCs may be in cross-talk with resident NCs and have a functional role in the differentiation and maintenance of NCs.

What is the role of NCs in the IVD degeneration process and hence their potential function in IVD regeneration? The NCs may have a dual regenerative role: they may instruct CLCs and/or replenish by differentiating into CLCs. NCs may serve as 'organizer’ cells, influencing the surrounding NP-cell homeostasis to maintain NP integrity. It seems reasonable to consider NCs as a potential source of growth factors that stimulate NP matrix synthesis [59-63], given the signalling role of the notochord during embryonic development [41]. NC-conditioned medium (NCCM; medium in which NCs or NC-containing tissue is cultured for 4 days and comprises NC-secreted soluble factors) has the potential to increase NP GAG production and cell proliferation [61-63]. Furthermore, both NCCM and CLC + NC co-culture stimulate the *in vitro* differentiation of MSCs into a NP-like phenotype with a high chondrogenic matrix production [36,39,42,59,60,64,65]. NCs may maintain the young NP phenotype by secreting factors that suppress cell death and by influencing genes that regulate IVD anabolic activity and matrix protection [66]. Attempts have been made to identify, isolate, and/or synthesize the bioactive factors in NCCM for use in therapeutic interventions [61]. The matrix proteins clusterin and tenascin, and connective tissue growth factor (CTGF) have been proposed to be the bioactive factors in NCCM [60,67,68]. Indeed, CTGF has been shown to promote matrix production, proliferation, differentiation, and cell migration and to inhibit apoptosis, effects that prevent the development of IVD degeneration [67-69].

However, NCs are also considered to be 'progenitors’ of the CLC population. It is much debated whether the chondrification of the maturing IVD is due to the differentiation of NCs into a CLC-like phenotype (stem cell function) or apoptosis of the NCs followed by invasion of CLCs from other locations, e.g. the EPs and/or inner AF [27,58].

There are several arguments for and against the notion that NCs are CLC progenitor cells. The first argument that disputes the progenitor function is the considerable difference in cell morphology and size of CLCs (17–23 μm) and NCs (25–85 μm) [1,8]. Secondly, NCs and NCCM are both able to stimulate the migration of EP chondrocytes [70], which makes the NC organizer function more likely than the progenitor function. Thirdly, two cell types have been detected in the developing notochord: large vacuolated cells and smaller non-vacuolated cells [71]. Thus, the presumed origin of NP tissue, i.e. the notochord, seems to contain cells that appear similar to those detected in the NP itself. Fourthly, the profile of gene expression is different in NCs and CLCs, which also opposes the progenitor function status of NCs [72]. Fifthly, the self-renewal of NCs occurs infrequently and is not unlimited, aspects that oppose the stem/progenitor cell function [58]. Lastly, *Brachyury* expression - often considered a NC marker and required for the differentiation of mesoderm into notochord [3] - was not detected in the human adult NP, which may argue against the hypothesis that both NC and CLCs are derived from the notochord [73]. The expression of this NC marker can, however, simply be lost during (trans) differentiation of NCs into CLCs.

In contrast, there are several arguments in favour of the NC progenitor theory. Firstly, NCs decrease and CLCs increase in number during ageing and disc degeneration, which could be explained by the transformation of NCs into CLCs [1]. Histological and automated live cell-imaging studies of degenerated IVDs show that NCs can differentiate into cells morphologically similar to CLCs [58,74]. Secondly, NCs have the ability to renew themselves *in vitro*[58]. Thirdly, chondrocyte volumes can vary about 10-fold in the growth plate, which counteracts the argument that the differences in cell size are too large for NCs to be a progenitor for CLCs. This variation in cell morphology and size can be explained by different maturation stages and/or cell function [1,3]. Porcine NCs have been shown to exhibit a significant variation in cell morphology, size, and number [75], implying that NC morphology can change with time. Fourthly, it is known from growth plate chondrocytes that, within a single cell population, cells of different sizes and/or location can express different gene profiles, which could explain the difference in gene expression profiles in NCs and CLCs [1,3]. In fact, the gene expression of NCs and CLCs showed substantial overlap, and *Brachyury* expression remained constant during IVD degeneration when NCs were replaced by CLCs [58,76]. Fifthly, NCs and CLCs have been shown to synthesize proteoglycans at the same rate [58], which indicates that they have a comparable capacity to produce matrix. In contrast, others found that NCs synthesized proteoglycans at significantly greater rates than CLCs [77]. However, in this study, the CD dogs (donors of the CLCs) were older and had a higher disc degeneration grade than the NCD dogs (donors of the NCs), which could have influenced the study outcomes. Sixthly, trace lineage studies indicate that both NCs and CLCs are derived from the embryonic notochord, suggesting that NCs are precursors of CLCs [78,79]. Lastly, there is no marker that is homogenously expressed within a specific population (NC or CLC) and, therefore, it is impossible to state that both cells of notochordal and mesenchymal origin are present within the IVD [1,3].

There are some points of interest concerning the design of experiments investigating NC-based regenerative strategies for the treatment of IVD degeneration. Some species of experimental animals retain NCs throughout life, whereas others lose them during development and ageing, which may considerably influence experimental outcomes. For example, most studies report that NCs are present in skeletally mature non-chondrodystrophic dogs, pigs, ferrets, rabbits, rats, and mice [4,5,8,41], whereas in chondrodystrophic dogs, goats, horses, cows, and sheep, they disappear before skeletal maturity is reached [4,5,8,80]. In contrast, others favor the hypothesis that in all animal species, including humans, NCs remain in the NP throughout life [3]. It is important that inter-species differences are taken into consideration and that research results are interpreted with care. Furthermore, NC isolation has the disadvantage of a low purity or yield [58], which is further complicated by the lack of specific markers for NCs [8]. *Brachyury*, and *Cytokeratin 8* or *19* have been proposed as NC markers [3,81,82] but they are not very specific and show interspecies variation [76]. Additional obstacles are the slow growth of NCs and their sensitivity to the external environment [58]. It is essential that culture conditions are optimized, so that NCs do not lose their characteristics. Thus far, culture media pH, glucose concentration, osmolality, and O_2_ and CO_2_ percentages have been shown to affect maintenance of the NC-phenotype in culture [69,83].

### Growth factors

Stimulation of cell proliferation and/or matrix synthesis with exogenous growth factors alters IVD homeostasis either by inhibiting catabolic and/or by stimulating anabolic processes [84]. Several growth factors have been tested *in vitro* and most have been shown to successfully decrease cell apoptosis and/or to enhance chondrogenic matrix production, and/or to direct MSC differentiation towards a NP-like phenotype. Growth factors have also been proven effective *in vivo* in animal models with experimentally induced IVD degeneration (Additional file 1). However, to our knowledge there have been no studies of growth factors tested in animal models with naturally occurring IVD degeneration.

The half-life and solubility of growth factors, the proper carrier, and the presence of inhibitors need to be taken into consideration when growth factors are used in regenerative strategies [84]. Since the NP is a confined, avascular space, the injected factor may be retained for long time. Some factors have been shown to bind to the ECM and metabolic changes following a single growth factor injection might be sustained, potentially leading to long-lasting effects [84]. If a growth factor has a short half-life, it can be administered by sustained delivery systems. Since degenerated discs contain relatively few cells, the injection of growth factors may not produce an optimal therapeutic effect [84]. Combined cell- and growth factor-based therapy may solve this problem.

In conclusion, a therapeutic approach to IVD degeneration involving enhanced tissue repair by exogenous growth factors may be clinically important, but more research is needed to address the efficacy, duration of action, safety, and adverse effects of the growth factors administered *in vivo*.

### Gene therapy

In gene therapy-based regenerative medicine, messenger RNA can be degraded to decrease the level of expression of relevant genes or exogenous genes can be inserted into cells by means of (non-) viral vectors to increase gene expression [85]. Once the transferred gene is functional, the genetically modified cells can produce the desired product (e.g. a specific growth factor) in a continuous fashion with long-lasting effects [86]. Major barriers to the clinical use of gene therapy, however, include transfection efficiency, the creation of long-term effects, the difficulty of controlling cell proliferation, ectopic transfection, disease transmission, patient-specific dose responses, immune reactions against viral proteins, possible side effects, and ethical issues [87,88]. For these reasons, gene therapy has not been used in a clinical setting yet.

## Drawbacks of regenerative treatment strategies for IVD degeneration

Regenerative treatment strategies have some drawbacks. Firstly, early identification of IVD degeneration is essential to prevent the occurrence of IVD disease. Early IVD degeneration can be detected by means of MRI studies, but in veterinary practise this diagnostic tool is typically only used when clinical signs are profound. In the case of early IVD degeneration with minor clinical signs, there is the ethical issue of whether the risks of an intradiscal injection are not higher than the benefit that may be expected from the novel regenerative treatment [89]. Secondly, several cell- and growth factor-based regenerative treatment strategies have been tested *in vitro* and *in vivo* in animal models with experimentally induced IVD degeneration. As CD breed dogs have proven a suitable animal model of naturally occurring IVD degeneration [9,13], we advocate using this model to assess the (side) effects of novel regenerative treatments. NP tissue from CD and NCD dogs show differences in gene expression profiles [14] and, therefore, future studies should aim at determining whether CD and NCD dogs also respond differently on IVD regenerative treatments. Thirdly, the performed regenerative IVD studies show halting of degeneration (by increased disc height, proteoglycan or water content, and improved clinical status of patients during follow-up), but it is not known whether the cell-based IVD treatment strategies were actually able to regenerate NP tissue. Therefore, a critical analysis of study results is important, for instance by assessing how close the regenerated IVD is compared with a healthy IVD. Fourthly, the severity of the IVD degeneration possibly influences the therapeutic effect of injected cells and/or growth factors, since degenerated CLCs may have lost their chondrogenic potential and the effect of injected cells and/or growth factors may differ between early and severely degenerated IVDs [90]. These aspects should also be investigated in future studies. Fifthly, all regenerative strategies based on injectable treatments may cause side effects. Cell leakage after intradiscal BMSC injection has been reported to induce osteophyte formation in a rabbit IVD degeneration model [91]. Furthermore, the dose of growth factor to be used needs to be fine-tuned. For example, intradiscal injection of bone morphogenetic protein-2 (BMP-2) induced degenerative changes [92] and weekly intradiscal treatment with growth and differentiation factor-5 (GDF-5) or insulin-like growth factor-1 (IGF-1) resulted in inflammatory reactions in the adjacent vertebrae and connective tissue infiltrates in the NP, causing the IVD to collapse [93]. The latter was also noted in IVDs injected with saline and may be caused by multiple needle insertions [93]. Also, misplacement of recombinant gene therapy products has been associated with lower extremity paralysis after intradural injection, and with paraesthesia, systemic illness, and death after epidural injection [94,95]. Furthermore, the sudden acceleration of the cell reproductive cycle by growth factor or gene therapy treatment has been linked with chromosomal translocations or tumorigenesis [32]. Lastly, a meta-analysis demonstrated that treatment of a degenerated IVD with cells and biomaterials showed better results on disc height restoration and MRI T2 signal intensity than treatment with cells or biomaterial alone. However, none of the treatments could fully restore the disc height or achieve the same MRI T2 signal intensity as the healthy disc [96].

## Current regenerative treatments in human patients

Nearly three-quarters of the human population will be affected by low back pain at some stage in their lives [23]. While this condition is multifactorial, IVD degeneration is one of its major causes, involved in at least 40% of chronic back pain cases [97]. As no effective therapies to retard or reverse disc degeneration have yet been devised, there is huge interest in potential regenerative treatments for human patients. Therefore, over the past few years, several human clinical phase I (safety) and II (efficacy) cell-based trials have been initiated.

In the Euro Disc study, CLCs were obtained from 112 human patients with IVD herniation who underwent discectomy, subsequently culture-expanded and after 12 weeks percutaneously re-injected into the IVD [23]. Interim analysis revealed that human patients receiving autologous CLC transplantation experienced a greater pain reduction after 2 years and had a higher IVD fluid content on MRI than patients who did not undergo CLC transplantation after discectomy [23]. Recently, ten human patients with lumbar IVD disease treated with an intradiscal BMSC injection showed diminished pain and disability and a significantly elevated IVD water content (MRI) at the 1-year follow-up [98]. The same research group is currently performing a clinical study on the treatment of IVD disease with allogeneic BMSCs in twenty-four human patients (http://clinicaltrials.gov/show/NCT01860417). Furthermore, another phase II clinical trial enrolled 100 human patients with a single painful, degenerated lumbar IVD. Interim analysis of 50% of the patients after 6 months follow-up indicated a greater reduction in low back pain and an improvement in function in patients receiving 6*10^6^ immunoselected and culture-expanded MSCs compared with control patients. The MRI status of the injected IVD, however, was not affected by the treatment. No cell-related safety issues were encountered in this trial, although the patients receiving 18*10^6^ MSCs showed a higher incidence of adverse effects (http://clinicaltrials.gov/show/NCT01290367).

Lastly, several human growth factor-based phase I/II clinical trials were started to evaluate the safety, tolerability, and preliminary effectiveness of intradiscal delivery of growth and differentiation factor-5 (GDF-5) as compared with placebo (sterile water/vehicle control) in human patients with early lumbar disc degeneration in the United States, Australia, and the Republic of Korea. Clinical and safety outcomes in these trials are the development of adverse events and the neurological status of patients (http://clinicaltrials.gov/show/: NCT00813813, NCT01124006, NCT01158924, and NCT01182337).

Over the past few years, both human and veterinary medicine have recognized the importance of the 'One Health’ concept: bringing together human and animal health for new medical solutions, advantageous for humans as well as animals. Dogs are the only animals that spontaneously develop IVD disease that is diagnosed and treated, both medically and surgically, in the same way as in humans. Our research group has previously shown that the gross pathology, histopathology, GAG content, and MMP-2 activity of human and canine IVDs were similar in all different stages of IVD degeneration [9]. Only some differences between human and canine IVDs were found, such as the absence of growth plates in developing human vertebrae, thicker cartilaginous endplates and more pronounced endplate irregularities in humans with increasing severity of IVD degeneration [9]. Although the canine IVDs were smaller than the human IVDs, the ratio of NP area/IVD area was similar in the two species [9]. These facts indicate that human and canine IVD degeneration is comparable at many different levels and that humans and dogs can benefit from each other by being a suitable model for the other species. Human and canine patients with IVD degeneration can thus be used as a study population to investigate the mechanisms of degeneration and potential regenerative treatments, which is beneficial for both species and suits the 'One Health’ concept.

## Conclusions

MSC-based regenerative treatment of the degenerated IVD seems promising. Clinical indications for intradiscal injection of MSCs are (a) treatment of discogenic pain without severe compression and/or neurologic deficits and (b) treatment of the adjacent degenerated discs during decompressive spinal surgery. Percutaneous intradiscal injection is technically feasible in dogs [21-23]. This altogether paves the way for prospective phase I-II clinical trials of minimally invasive cell- or growth factor-based treatment in canine patients with IVD degeneration and disease.

New strategies concentrate on enhancing the regenerative potential of MSCs by growth factors, including those that are specifically secreted by the NCs. Moreover, the conditions under which the regenerative treatment may achieve maximal MSC and resident NP cell performance - even in advanced stages of IVD degeneration - could be improved by creating an optimal biomechanical environment.

## Abbreviations

AF: Annulus fibrosus; ASC: Adipose-derived stem cell; BMP-2: Bone morphogenetic protein-2; BMSC: Bone marrow-derived stem cell; CD: Chondrodystrophic; CLC: Chondrocyte-like cell; CTGF: Connective tissue growth factor; DH: Disc height; ECM: Extracellular matrix; EP: End plate; GAG: Glycosaminoglycan; GDF-5: Growth and differentiation factor-5; IGF-1: Isulin-like growth factor-1; IVD: Intervertebral disc; MSC: Mesenchymal stromal (stem) cells; MMP: Matrix metalloproteinase; MRI: Magnetic resonance imaging; NC: Notochordal cell; NCCM: Notochordal cell conditioned medium; NCD: Non-chondrodystrophic; NP: Nucleus pulposus; NPPC: Nucleus pulposus progenitor cell.

## Competing interests

The authors declare that they have no competing interests.

## Authors’ contributions

FB mined and analyzed the data, participated in the design of the review, and drafted the manuscript. NW and LP assisted in drafting the manuscript. KI participated in the design of the review and helped to draft the manuscript. BM and MT conceived the study, helped to draft the manuscript, and coordinated the process. All authors read and approved the final manuscript.

## Supplementary Material

Additional file 1 **Growth factors tested *in vivo *** **in animal models with experimentally induced intervertebral disc (IVD) degeneration.** Click here for file
