# Supplementary material for: Potential regenerative treatment strategies for intervertebral disc degeneration in dogs
Source: BMC Vet Res. 2014 Jan 4;10:3. doi: 10.1186/1746-6148-10-3 (PMC3914844; doi:10.1186/1746-6148-10-3)
Supplement: Additional file 1 — Growth factors tested in vivo in animal models with experimentally induced intervertebral disc (IVD) degeneration. [file 1746-6148-10-3-S1.pdf]

| Growth factors tested *in vivo* in animal models with experimentally induced intervertebral disc (IVD) degeneration | | | | | | |
|---|---|---|---|---|---|---|
| **Growth factor** | **Mechanism of action** | **Animal model Induction of degeneration** | **Treatment dose; duration Control treatment** | **GAG/PG content of the NP** | **Histo(patho)logy** | **MRI/radiograph** |
| **BMP-2** | Differentiation signalling factor; anabolic | 17 rabbits [1] AF tearing | 0.1 mg (in 0.1 mL); 12 wks Control: saline | ND | Vascularity, fibroblast proliferation, inflammation | Degenerative changes; disc space narrowing/irregularity, subchondral sclerosis, osteophytes, EP hypertrophy |
| **BMP-7/OP-1** | Differentiation signalling factor; anabolic | 34 rats [2] Compression | 0.2 µg (in 1 µL); 4 wks Control: saline | ND | ECM content + (NS) NP size + | ND |
| | | 90 rabbits [3] AF puncture | 100 µg (in 10 µL); 2, 4, 8, 12, 24 wks Control: lactose | + | Total histological scores (AF fibers, NP cellularity and matrix) + | DH + MRI score + MRI T2W intensity + (NS) |
| | | 16 rabbits [4] AF puncture | 100 µg (in 10 µL); 8 wks Control: lactose | + | ND | DH + MRI T2W intensity + |
| | | 54 rabbits [5] Chondroitinase | 100 µg (in 10 µL); 6, 8, 12, 16 wks Control: lactose | + | Some BMP-7-treated IVDs with abundant matrix | DH + |
| | | 24 rabbits [6] None | 2 µg (in 10 µl); 2, 4, 8 wks Control: saline | + (only significant at 2 wks) | ND | DH + |
| **BMP-14/GDF-5** | Differentiation signalling factor; anabolic | 16 mice [7] Compression | 8 ng (in 8 µL); 1, 4 wks Control: saline | ND | DH + PC + (NS) Collagen 2, aggrecan expression (NP) | ND |
| | | 16 rabbits [8] AF puncture | 10 ng, 1 or 100 µg (in 10 µl); 12 wks Control: puncture alone, puncture + PBS | ND | Total histological score (AF fibers, NP cellularity and ECM) at 100 µg + (vs. puncture alone) | DH + (only significant at 1 and 100 µg) MRI scores + (all doses, NS) MRI T2W intensity + (all doses) |
| **FGF** | Signalling factor; anabolic | 10 mice [7] Compression | 8 ng (in 8 µL); 1, 4 wks Control: saline | ND | Cell density in middle/inner AF + (NS) | ND |
| **IGF-1** | Insulin-like activity; anabolic | 16 mice [7] Compression | 8 ng (in 8 µL); 1, 4 wks Control: saline | ND | DHI, inner annular fibrochondrocytes, and PC + (all NS) | ND |
| **Link N** | Peptide, stabilizing PG aggregates | 28 rabbits [9] AF puncture | 100 µg (in 10 µl); 12 wks Control: saline | + (NS) | Less signs of IVD degeneration (NS) | DH + |
| **P2K** | Peptide, possibly regulating TGFβ signalling | 14 rabbits [10] AF puncture | 10 µg (in 15 µL); 12 wks Control: lactose | + | Less signs of IVD degeneration ECM content + | DH + MRI scores + MRI T2W intensity + |
| **PRP** | Plasma fraction with highly concentrated growth factor levels | 36 rabbits [11] NP aspiration | 20 µL gelatin hydrogel microspheres (GHM) impregnated with PRP (sustained release); 2, 4, 8 wks Control: PBS-GHM | ND | Less signs of IVD degeneration PG expression + (NS) | ND |
| | | 128 rabbits [12] NP aspiration | 20 µL GHM impregnated with PRP (sustained release); 2, 4, 8 wks Control: PBS-GHM | ND | Less apoptotic NP cells | DH + MRI scores + MRI T2W intensity + (NS) |
| | | 12 rabbits [13] AF puncture | 20 µL PRP; 12 wks Control: PBS | ND | CLC number + Histological scores not different | DH + MRI T2W intensity + (NS) |
| **Simvastatin** | Stimulates BMP-2 pathway *in vitro* | 30 rats [14] AF puncture | PEG gel with 10 µg simvastatin; 2 wks Control: PEG gel | + (NS) | Total histological scores (AF fibers, NP cellularity and ECM) + | MRI scores + MRI T2W intensity + |
| **TGFβ** | Signalling factor; anabolic | 19 mice [7] Compression | 1.6 ng (in 8 µL); 1, 4 wks Control: saline | ND | PC + (NS) | ND |

Additional file 1. Growth factors tested *in vivo* in animal models with experimentally induced intervertebral disc (IVD) degeneration.
+: significantly better/higher than in the control group; AF, annulus fibrosus; BMP, bone morphogenetic protein; DH, disc height; ECM, extracellular matrix; EP, end plate; FGF, fibroblast growth factor; GAG, glycosaminoglycan; GDF-5, growth and differentiation factor-5; GHM, gelatin hydrogel microspheres; IGF-1, insulin-like growth factor 1; MRI, magnetic resonance imaging; ND, not done; NP, nucleus pulposus; NS, not significant; OP-1, osteogenic protein-1; P2K, Peniel 2000; PC, percentage proliferating cells; PG, proteoglycan; PRP, platelet rich plasma; TGFβ, transforming growth factor-β; T2W, T2-weighted; wks, weeks.

**References**


1. Huang KY, Yan JJ, Hsieh CC, Chang MS, Lin RM: **The in vivo biological effects of intradiscal recombinant human bone morphogenetic protein-2 on the injured intervertebral disc: an animal experiment.** *Spine (Phila Pa 1976)* 2007, **32**(11):1174-1180.
2. Kawakami M, Matsumoto T, Hashizume H, Kuribayashi K, Chubinskaya S, Yoshida M: **Osteogenic protein-1 (osteogenic protein-1/bone morphogenetic protein-7) inhibits degeneration and pain-related behavior induced by chronically compressed nucleus pulposus in the rat.** *Spine (Phila Pa 1976)* 2005, **30**(17):1933-1939.
3. Masuda K, Imai Y, Okuma M, Muehleman C, Nakagawa K, Akeda K, Thonar E, Andersson G, An HS: **Osteogenic protein-1 injection into a degenerated disc induces the restoration of disc height and structural changes in the rabbit anular puncture model.** *Spine (Phila Pa 1976)* 2006, **31**(7):742-754.
4. Miyamoto K, Masuda K, Kim JG, Inoue N, Akeda K, Andersson GB, An HS: **Intradiscal injections of osteogenic protein-1 restore the viscoelastic properties of degenerated intervertebral discs.** *Spine J* 2006, **6**(6):692-703.
5. Imai Y, Okuma M, An HS, Nakagawa K, Yamada M, Muehleman C, Thonar E, Masuda K: **Restoration of disc height loss by recombinant human osteogenic protein-1 injection into intervertebral discs undergoing degeneration induced by an intradiscal injection of chondroitinase ABC.** *Spine (Phila Pa 1976)* 2007, **32**(11):1197-1205.
6. An HS, Takegami K, Kamada H, Nguyen CM, Thonar EJ, Singh K, Andersson GB, Masuda K: **Intradiscal administration of osteogenic protein-1 increases intervertebral disc height and proteoglycan content in the nucleus pulposus in normal adolescent rabbits.** *Spine (Phila Pa 1976)* 2005, **30**(1):25-31; discussion 31-2.
7. Walsh AJ, Bradford DS, Lotz JC: **In vivo growth factor treatment of degenerated intervertebral discs.** *Spine (Phila Pa 1976)* 2004, **29**(2):156-163.
8. Chujo T, An HS, Akeda K, Miyamoto K, Muehleman C, Attawia M, Andersson G, Masuda K: **Effects of growth differentiation factor-5 on the intervertebral disc--in vitro bovine study and in vivo rabbit disc degeneration model study.** *Spine (Phila Pa 1976)* 2006, **31**(25):2909-2917.
9. Mwale F, Masuda K, Pichika R, Epure LM, Yoshikawa T, Hemmad A, Roughley PJ, Antoniou J: **The efficacy of Link N as a mediator of repair in a rabbit model of intervertebral disc degeneration.** *Arthritis Res Ther* 2011, **13**(4):R120.
10. Kwon YJ, Lee JW, Moon EJ, Chung YG, Kim OS, Kim HJ: **Anabolic effects of Peniel 2000, a peptide that regulates TGF-beta1 signaling on intervertebral disc degeneration.** *Spine (Phila Pa 1976)* 2013, **38**(2):E49-58.
11. Nagae M, Ikeda T, Mikami Y, Hase H, Ozawa H, Matsuda K, Sakamoto H, Tabata Y, Kawata M, Kubo T: **Intervertebral disc regeneration using platelet-rich plasma and biodegradable gelatin hydrogel microspheres.** *Tissue Eng* 2007, **13**(1):147-158.
12. Sawamura K, Ikeda T, Nagae M, Okamoto S, Mikami Y, Hase H, Ikoma K, Yamada T, Sakamoto H, Matsuda K, Tabata Y, Kawata M, Kubo T: **Characterization of in vivo effects of platelet-rich plasma and biodegradable gelatin hydrogel microspheres on degenerated intervertebral discs.** *Tissue Eng Part A* 2009, **15**(12):3719-3727.
13. Obata S, Akeda K, Imanishi T, Masuda K, Bae W, Morimoto R, Asanuma Y, Kasai Y, Uchida A, Sudo A: **Effect of autologous platelet-rich plasma-releasate on intervertebral disc degeneration in the rabbit anular puncture model: a preclinical study.** *Arthritis Res Ther* 2012, **14**(6):R241.
14. Zhang H, Wang L, Park JB, Park P, Yang VC, Hollister SJ, La Marca F, Lin CY: **Intradiscal injection of simvastatin retards progression of intervertebral disc degeneration induced by stab injury.** *Arthritis Res Ther* 2009, **11**(6):R172.
